# Capillary hemangioma as a rare benign tumor of the oral cavity: a case report

**DOI:** 10.4076/1757-1626-2-8622

**Published:** 2009-09-09

**Authors:** Alparslan Dilsiz, Tugba Aydin, Nesrin Gursan

**Affiliations:** 1 Department of Periodontology, Faculty of Dentistry, Ataturk University, 25240, Erzurum/Turkey; 2 Department of Periodontology, Faculty of Dentistry, Ataturk University, 25240, Erzurum/Turkey; 3 Department of Pathology, Faculty of Medicine, Ataturk University, 25240, Erzurum/Turkey

## Abstract

**Introduction:**

Hemangioma is a relatively common benign proliferation of blood vessels that primarily develops during childhood. Two main forms of hemangioma recognized: capillary and cavernous. The capillary form presents as a flat area consisting of numerous small capillaries. Cavernous hemangioma appears as an elevated lesion of a deep red color, and consists of large dilated sinuses filled with blood. The purpose of the study was to report the case of a capillary hemangioma in a patient and to describe the successful treatment of this case.

**Case presentation:**

The patient was a 19-year-old female who presented herself to the Atatürk University, Faculty of Dentistry, Department of Periodontology, with the complaint of bleeding and slowly enlarging mass on the upper right molar region. The lesion was diagnosed as capillary hemangioma after clinical examination and biopsy. Treatment consisted of scaling, root planning and surgical excision. Four months after surgery healing was occurred and two years later area of the lesion appeared completely normal as clinically.

**Conclusions:**

The surface is highly keratinized and no further growth was evidenced during the two year of follow-up. Early detection and biopsy is necessary to determine the clinical behavior of the tumor and potential dentoalveolar complications.

## Introduction

A number of terms have been used to describe vascular lesions, which are classified either as hemangiomas or vascular malformations [1]-[3]. Hemangioma is a term that encompasses a heterogeneous group of clinical benign vascular lesions that have similar histologic features [2]. It is bening lesion, which is a proliferating mass of blood vessels and do not undergo malignant transformation. There is a higher incidence in females than males. Although a few cases are congenital, most develop in childhood [2]. Occasionally, older individuals are affected [2,3]. The congenital hemangioma is often present at birth and may become more apparent throughout life [2].

Althought hemangioma is considered one of the most common soft tissue tumors of the head and neck [2], it is relatively rare in the oral cavity and uncommonly encountered by the clinicians. They may be cutaneous, involving skin, lips and deeper structures; mucosal, involving the lining of the oral cavity; intramuscular, involving masticator and perioral muscles; or intra-osseous, involving mandible and/or maxilla [4,5].

Hemangiomas are also classified on the basis of their histological appearance. Capillary and cavernous hemangiomas are defined according to the size of vascular spaces [2,6]. Capillary hemangioma are composed of small thin-walled vessels of capillary size that are lined by a single layer of flattened or plump endothelial cells and surrounded by a discontinuous layer of pericytes and reticular fibres [6]. To our knowledge, it was first described in the literature by Sznajder et al. [7], in 1973 under the term "Hemorrhagic hemangioma". Cavernous hemangiomas consist of deep, irregular, dermal blood-filled channels [2]. They are composed of tangles of thin-walled cavernous vessels or sinusoids that are separated by a scanty connective tissue stroma [6]. Mixed hemangiomas contain both components and may be more common than the pure cavernous lesions [6].

Clinically hemangiomas are characterized as a soft mass, smooth or lobulated, sessile or pedunculated and may be seen in any size from a few millimeters to several centimeters [6]. The color of the lesion ranges from pink to red purple and tumor blanches on the application of pressure, and hemorrhage may occur either spontaneously or after minor trauma. They are generally painless. These tumors are mostly seen on the face, fingers and occasionally seen on oral mucosa. Oral hemangiomas are usually seen on the gingiva and less frequently at other sites where it occurs as a capillary or cavernous type, more commonly the former [6]. Periodontally, these lesions often appear to arise from the interdental gingival papilla and to spread laterally to involve adjacent teeth [8].

Management of hemangiomas and the treatment of choice depend on several factors including the age of the patient and the size and extent of the lesions, as well as their clinical characteristics. Some congenital lesions may undergo spontaneous regression at an early age [9]. If superficial lesions are not an esthetic problem and are not subject to masticatory trauma, they may be left untreated [3]. Small and superficial lesions may be completely excised with relative ease. However, excision of more deeply seated lesions usually involves a wider surgical approach, which may result in a disfigurement that is difficult to accept for the treatment of these lesions. In addition, emergency surgery may become mandatory when arterial bleeding arises from intraosseous hemangiomas of the jaw following simple tooth extraction [4].

Various treatments have been used in the management of hemangiomas, including oral corticosteroids, intralesional injection of fibrosing agents, interferon α-2b, radiation, electrocoagulation, cryosurgery, laser therapy, embolization and surgical excision [11]-[13]. Recurrence has been reported [1,2].

The purpose of the study was to report the case of a capillary hemangioma in a patient and to describe the successful treatment of this case.

## Case presentation

In November 2000, a 19-year-old Turkish female was referred by her dentist to the Department of Periodontology of the Faculty of Dentistry, Ataturk University, for evaluation and treatment of the gingival bleeding and overgrowth.

According to the patient, she suffered from excessive gingival bleeding during meals that had started four months ago, accompanied by elevated gingival reddish. A short time later, she discovered a dark red swelling on her upper right gingival tissues. The swelling in the associated region had been increasing gradually since that time. She did not give any relevant past dental history. The patient's medical history was non-contributory and she did not take any medications. She and her parents stated that, in March 1990 and June 2000, she had a lesion operated and diagnosed as congenital hemangioma on the right of her face.

**Figure 1:**
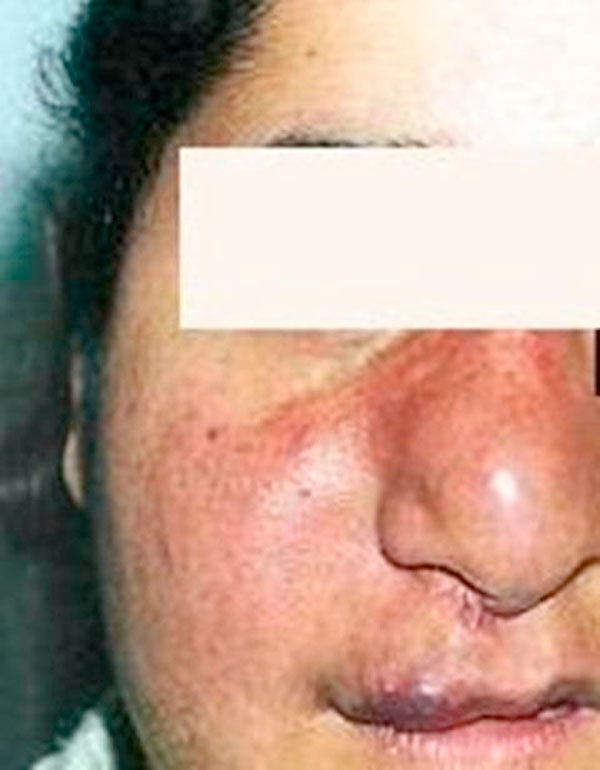
**Clinical aspect of the congenital hemangioma on the patient's face in November 2000**.

During physical examination (Figure 1), a right-sided hemihypertrophy of the face with congenital hemangioma was observed. Her mouth was deviated toward the left side of her face. No other similar lesions were clinically visible in the head and neck region. Moreover, no lymph nodes were palpable.

**Figure 2:**
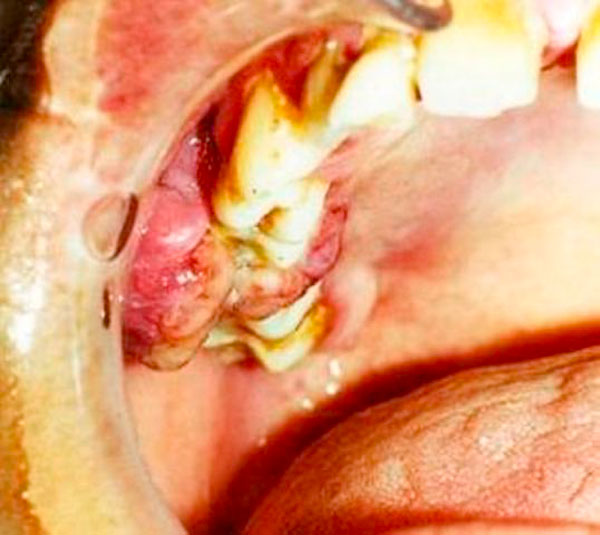
**Clinical view of the Capillary hemangioma**.

Clinical evaluation revealed a mass on the buccal surface of upper right molar region (Figure 2). It was firm, pedunculated and red mass, and was located in the attached gingiva in the right maxillary region, covering almost the entire coronal part of the #3 and #4 teeth. On the palatal side the mass extended throughout the marginal and attached gingival of the second premolar and first molar. The mass was painful and bled easily upon palpation. Tooth #4 involved by the mass was mobile and a diastema had formed between #3 and #4 teeth. Periodontal pocket (approximately 10 mm) was detected in the associated region. Periodontal examination revealed a moderate and generalized gingivitis due to bacterial plaque. There was a mild accumulation of dental plaque and the gingival tissues were swollen. Other findings included a mild supragingival calculus around her teeth, absence of carious lesions and tooth malpositioning. It was provisionally diagnosed as a pyogenic granuloma.

**Figure 3:**
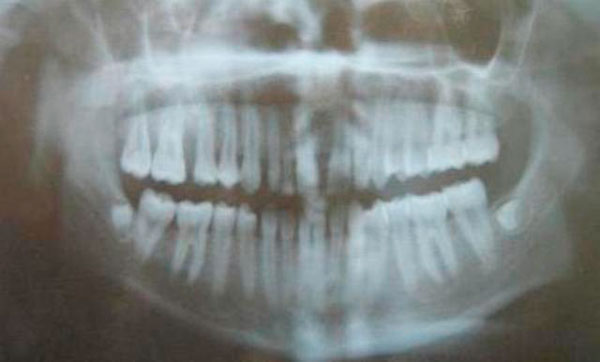
**OPG showing interdental diestema and localized bone loss in the regions of teeth #3 and #4**.

An orthopantomograph radiograph demonstrated that there was localized crestal bone destruction in the area of the tumor, missing tooth germs in upper third molars (Figure 3).

**Figure 4:**
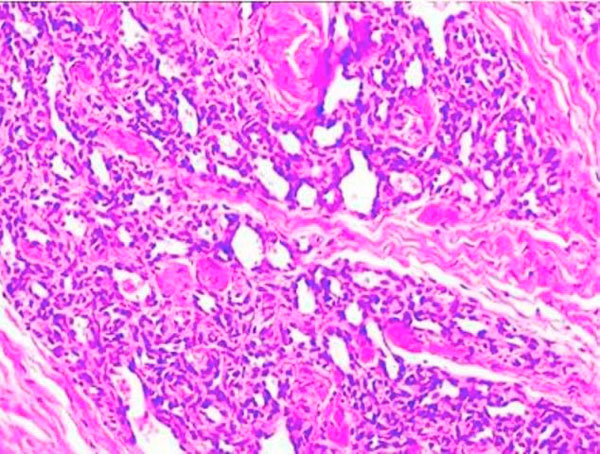
**Histopathological specimen; Capillary lumen formations in the deep connective tissue (×40 magnification, H-E staining)**.

A gingival biopsy was taken from the tumor zone, producing profuse hemorrhage controlled by pressure with gauze. The biopsy tissue was rinsed in formalin (10%), and sent for histopathologic examination. Histopathologic examination of the excised tissue revealed nonceratinize stratified squamous epithelium overlying on unencapsulated tumor composed of many thin-walled capillary channels. The capillaries were lined by a single layer of endothelial cells. Some areas showed marked endothelial cell proliferation. Sparse plasma cells and lymphocytes were seen scattered throughout stroma (Figure 4).

After having undergone clinical and physical examinations and laboratory evaluation in the pathology department, she was diagnosed as having capillary hemangioma.

Periodontal therapy consisted of oral hygiene instruction, full-mouth scaling and root planning, and modified Widman flap surgery.

Written informed consent was obtained from the patient after all treatment procedures had been fully explained.

### Periodontal management

Before surgical treatment of the tumor, a thorough scaling and root planning were done carefully to remove any local irritating factors that may have been responsible for the gingival inflammation. The patient was educated regarding good oral hygiene maintenance practices.

**Figure 5:**
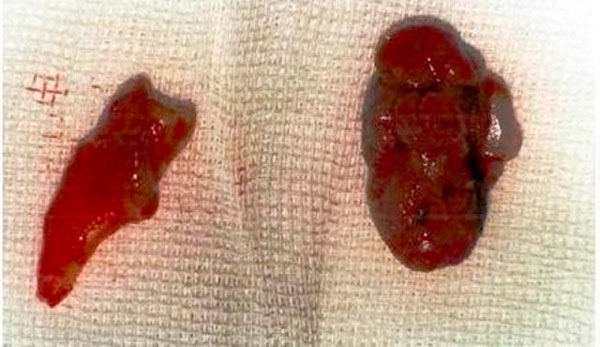
**The view of excised tissue and extracted tooth #4**.

**Figure 6:**
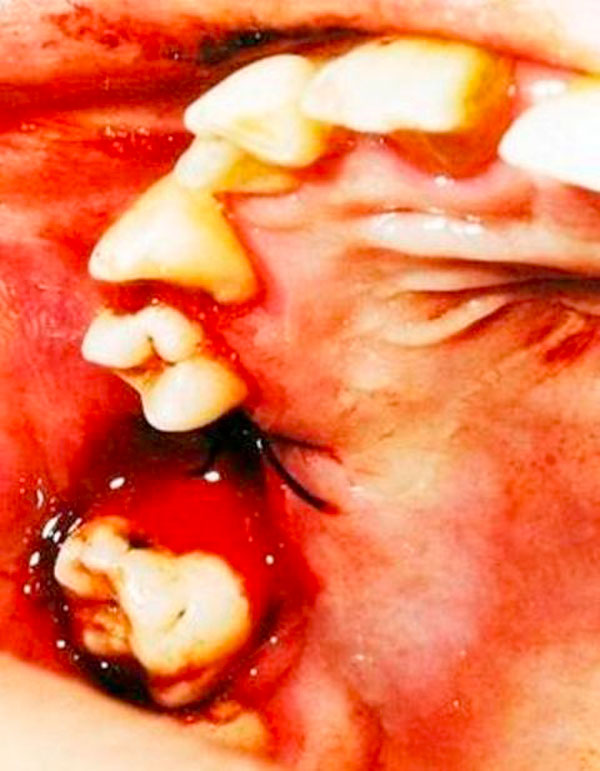
**Immediate postoperative view following periodontal surgery**.

Periodontal surgery was done under strict aseptic conditions using local anesthesia. The modified Widman flap surgical procedure was performed as described by Ramfjord and Nissle [14]. Initial incision was performed in the regions of teeth #2 through #5. The tumor was carefully removed the completely with the remaining granulation tissue after elevating buccal and palatal flaps, and tooth #4 had a poor prognosis, and it was extracted to eliminate the focus of infection (Figure 5). There was profuse intraoperative bleeding that was controlled with the help of pressure packs. The flaps were sutured with 3-0 non-resorbable silk sutures (Figure 6). The excised tissue was kept in formalin (10%) and sent for histopathologic examination. The histology was similar to that seen in the first specimen.

The patient was prescribed analgesics (Naproxen^£^ 550 mg, every 12 hours, 5 days) and instructed to rinse twice daily with 0.12% chlorhexidine rinse for 2 weeks postoperatively and to avoid trauma or pressure at the surgical site. Toothbrushing activities in the operated sites were discontinued during this time. The sutures were removed 7 days after surgery, home care instructions were given. Professional prophylaxis was done weekly for the first month and then at 4-month interval.

### Clinical observations

**Figure 7:**
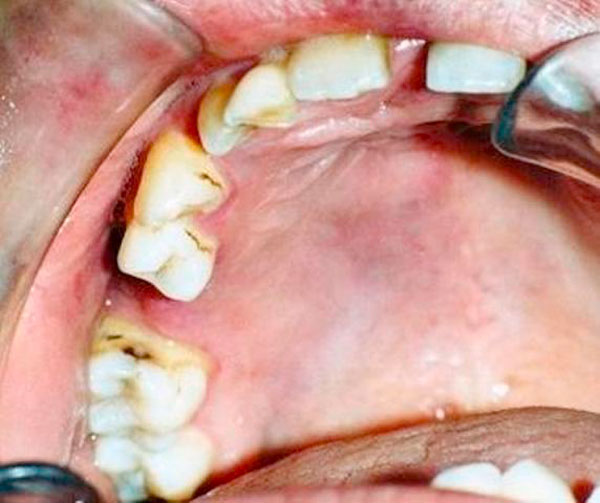
**Postoperative result 2 years following surgical periodontal therapy**.

**Figure 8:**
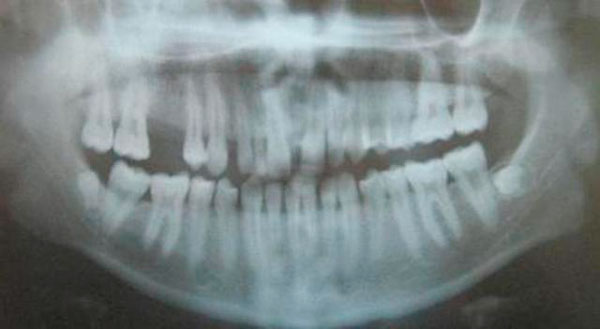
**Postoperative OPG view two years after surgical periodontal therapy**.

Four months following surgery, the affected area had completely healed, and there were no complications. Probing depth in the associated region was less than 2 mm. The patient's plaque control was good, although moderate tooth staining was apparent. The patient was periodically observed until two years after our treatment began. At that time there were no clinical or radiographic signs of recurrence (Figure 7 and 8). The patient was scheduled to receive a prosthetic replacement for tooth #4. ^&^Ultracaine DS Forte^®^, Hoechst Roussel, Frankfurt, Germany. ^£^Apranax^®^, Abdi Ibrahim Drug Ltd., Istanbul, Turkey. Kloroben^®^, Drogsan Drug Ltd., Istanbul, Turkey.

## Discussion

Hemangiomas are a common soft tissue tumor that often congenital or develop in the neonatal period and grow rapidly. They usually cover a large site, may be macular or raised and usually resolve progressively in childhood [2,3]. They may occur in the oral and maxillofacial region including gingiva, palatal mucosa, lips, jawbone, and salivary glands [1,5,7,10,15,16]. Apart from the oral cavity, capillary hemangioma developed at other sites such as eyelid, cheek and cauda equine were reported [1,17]. The patient in this case report had a congenital vascular lesion of face, was diagnosed capillary hemangioma, but there were no similar lesions of the other sites on the body.

The occurrence of hemangioma with its primary location on gingival tissues seems to be extremely rare. There are many clinical features of capillary hemangioma such as asymmetry of the face, spontaneous bleeding, pain, mobility of teeth, blanching of tissue, pulsation, expansion of bone, paresthesia, early exfoliation of primary teeth, delayed eruption, root resorption, and missing teeth [1,4,5,7,16]. In the case presented here, the patient has facial asymmetry, spontaneous bleeding, pain, mobility of tooth #4, blanching of tissue, and missing teeth.

Hemangiomas may mimic other lesions clinically, radiographically and histopathologically. The differential diagnosis of hemangiomas includes pyogenic granuloma, chronic inflammatory gingival hyperplasia (epulis), epulis granulomatosa, varicocell, talengectasia, and even with squamous cell carcinoma. The most common vascular proliferation of the oral mucosa is the pyogenic granuloma. This is a reactive lesion that develops rapidly, bleeds easily and is usually associated with inflammation and ulceration. Clinically, it is often lobulated, pedunculated and red to purple and it may be hormone sensitive [6]. There are two histological types of pyogenic granuloma of the oral cavity: the LCH and non-LCH type. LCH is characterized by proliferating blood vessels that are organized in lobular aggregates although superficially the lesion frequently undergoes no specific change, including edema, capillaries dilation or inflammatory granulation tissue reaction, whereas the second type consists of highly vascular proliferation that resembles granulation tissue [6,18]. Histopathologically, the capillary hemangioma exhibits a progression from a densely cellular proliferation of endothelial cells in the early stages to a lobular mass of well-formed capillaries in the mature phase, often resembling the pyogenic granuloma without the inflammatory features [2]. The present case has clinical features of a pyogenic granuloma, but has not microscopic features of pyogenic granuloma. Therefore, biopsy of tissue specimens is often necessary for definitive diagnosis of hemangiomas. In the case reported here, histopathological evaluation was made before and after surgical removed, and the findings correlated.

In addition, hemangiomas may be confused with the vascular-appearing lesions of the face or oral cavity, which may also represent the Sturge-Weber syndrome [19]. They are often located in the territory of the branches of the trigeminal nerve. Usually, they do not undergo spontaneous involution like hemangiomas do. Ocular and cerebral vascular lesions may be found in such cases. These lesions may be further classified into flat, telangiectatic, stellar and senile variants [6].

Precise diagnosis of the type of vascular lesion is important because it may influence treatment considerably. Angiographic studies are not strictly demonstrated for diagnosis of hemangiomas, and are utilized only to define the size and the extent of the lesion [1,16]. These are more complicated procedures than histopathological evaluation, have a higher morbidity, and may cause undesirable side-effects. For these reasons, no attempt to use angiography was made in this case. CT and MRI of these lesions have more recently been demonstrated, and have been successfully utilized for the diagnosis of hemangiomas, as for other lesions of soft tissues [19,20].

In the case presented here, treatment of the capillary hemangioma was done surgical periodontal treatment. The treatment of capillary hemangiomas varies considerably depending on the clinical features and the anatomic considerations. Surgical excision is generally the treatment of choice for capillary hemangioma [1,4,15,16]. For those lesions not amenable to surgery, other therapy such as intralesional injection of fibrosing agents, interferon α-2b, radiation, electrocoagulation, cryosurgery, laser therapy, embolization may be used [1,11,12].

Attempts to remove hemangiomas using surgical excision may lead to serious medical problems such as heavy bleeding. In addition, postoperative recurrence may encounter [1,4,7]. The case described here demonstrates that there has been no subsequent hemorrhage or other evidence of recurrence.

The present case is of periodontal interest in view of the onset of the lesion on the gingival tissue, as well as the conservative treatment used.

## Conclusions

Early detection and biopsy is necessary to determine the clinical behavior of the tumor and potential dentoalveolar complications. Althought a rare bening tumor of the oral cavity, capillary hemangioma is important to the periodontist because of its associated gingival vascular features and complications in the form of impaired nutrition and oral hygiene, increased accumulation of plaque and microorganisms, and increased susceptibility to oral infections, which can impair the systemic health of the affected individual. In addition, the periodontal surgical management of hemangiomas should be performed with caution because the tissues may bleed profusely intraoperatively and postoperatively.

## Abbreviations

CT: computerized tomography; LCH: lobular capillary hemangioma; MRI: magnetic resonance imaging.

## Consent

Written informed consent was obtained from the patient for publication of this case report and accompanying images. A copy of written consent is available for review by Editor-In-Chief of this Journal.

## Competing interests

The authors declare that they have no competing interests.

## Authors' contributions

AD conceived the idea of the report and wrote the manuscript. AD and TA performed local excision of the tumor with periodontal surgery. NG made the pathological diagnosis and performed histopathologic evaluation. All authors read and approved final version of manuscript.
